# Tumour Necrosis Factor α Inhibitors during Spondylarthritis: Therapeutic Maintenance, Reasons, and Predictive Factors of Discontinuation (Data from RBSMR Registry)

**DOI:** 10.31138/mjr.271223.ttm

**Published:** 2024-12-31

**Authors:** Oumaima Idrissi Ouali, Salma Mikou, Imane El Mezouar, Nessrine Akasbi, Moncef Maiouak, Ihsane Hmamouchi, Redouane Abouqal, Ahmed Bezza, Fadoua Allali, Rachid Bahiri, Imane El Bouchti, Imad Ghozlani, Hasna Hassikou, Linda Ichchou, Saadia Janani, Radouane Niamane, Abdellah El Maghraoui, Taoufik Harzy

**Affiliations:** 1 Rheumatology, Faculty of Medicine, Pharmacy and Dental Medicine, Sidi Mohamed Ben Abdellah University, Fez, Morocco.; 2 Faculty of Medicine, Pharmacy and Dental Medicine, Hassan II University Hospital, Epidemiology, Fez, Morocco,; 3 Faculty of Medicine, Health Sciences Research Centre (CReSS), International University of Rabat (UIR), Rabat, Morocco,; 4 Laboratory of Biostatistical, Clinical and Epidemiological Research, Rabat, Morocco,; 5 Military Hospital Mohammed V, Ibn Sina University Hospital, Rheumatology, Rabat, Morocco,; 6 El Ayachi Hospital, Ibn Sina University Hospital, Rheumatology B, University Mohamed V, Rabat, Morocco,; 7 El Ayachi Hospital, Ibn Sina University Hospital, Rheumatology A, University Mohamed V, Rabat, Moroccom; 8 Arrazi University Hospital, Rheumatology, Mohammed VI University Hospital, Marrakech, Morocco,; 9 Faculty of Medicine and Pharmacy, University Ibn Zohr, Rheumatology, Agadir, Morocco,; 10 Military Hospital Moulay Ismail, Hassan II University Hospital, Rheumatology, Meknes, Morocco,; 11 Mohammed VI University Hospital, Mohammed I University, Rheumatology, Oujda, Morocco,; 12 Ibn Rochd University Hospital, Rheumatology, Casablanca, Morocco,; 13 Military Hospital Avicenne, Rheumatology, Mohammed VI University Hospital, Marrakech, Morocco,; 14 Private Medical Office, Rheumatology, Rabat, Morocco,; 15 Rheumatology, University Mohamed V, Rabat, Morocco

**Keywords:** tumour necrosis factor α inhibitors, therapeutic maintenance, predictive factors of discontinuation, reasons, register, spondyloarthritis

## Abstract

**Objectives:**

The aim of this study was to study the therapeutic maintenance of tumour necrosis factor α inhibitors (TNFα-I) for spondyloarthritis patients enrolled in the Moroccan biotherapy registry and to analyse the reason and the predictive factors of stopping TNFα-I.

**Methods:**

Data were collected from a historical-prospective multicentre registry of adult patients with spondyloarthritis, treated with biological treatment, in the 10 rheumatology departments in Morocco. Maintenance was defined as the interval between the introduction and the suspension of the same TNFα-I.

**Results:**

190 patients under TNFα-I were included, their average age was 40.2 +/−13.6 years. The male gender predominated. On average, the duration of the disease was 11.7 years +/−6.7 years. The ongoing therapeutic maintenance of all TNFα-I drugs in our study was relatively high and exhibited a decline over time, decreasing from 63.8% at 1 year to 45% at 3 years. At the 36-month follow-up, 27.7% had stopped their treatment. It was found that taking nonsteroidal anti-inflammatory drugs (NSAIDs) between visits and having a high average Ankylosing Spondylitis Disease Activity Score with C-reactive protein on the day of the visit were identified as predictive factors for therapeutic discontinuation in multivariate analysis.

**Conclusion:**

The therapeutic maintenance level of TNFα-I in our study was satisfactory and comparable to other series. our study provides a more comprehensive understanding of the factors that contribute to the improved maintenance of treatment with TNFα-I. It delves into the reasons influencing treatment continuity and identifies predictive factors of discontinuation.

## INTRODUCTION

Spondyloarthritis (SpA) comprises a diverse group of chronic inflammatory rheumatic musculoskeletal diseases,^1^ with the potential for significant disability. It is characterised by inflammation in the spine’s entheses and involvement of the sacroiliac joints, resulting in lower back and buttock pain. Peripheral manifestations of SpA may affect the plantar fascia, leading to heel pain, as well as large joints in the lower limbs, primarily presenting as asymmetrical oligoarthritis. Specific extra-articular manifestations associated with SpA, include conditions like psoriasis, inflammatory bowel disease (IBD), and uveitis. Furthermore, SpA has a distinct genetic component, with the HLA-B27 gene being a prominent genetic marker, and it tends to be associated with a propensity for ankylosis.^2,3^

Synthesised by macrophages or monocytes during episodes of acute inflammation, tumour necrosis factor α (TNF-α) is a proinflammatory cytokine that plays a crucial role in various cellular signalling pathways.^4^ It is a significant component of the inflammatory cascade, associated with multiple forms of inflammation, and acts as a key contributor to the end-stage of the inflammatory process. It is also recognised as an initiator of inflammation and is implicated in numerous cutaneous and systemic inflammatory diseases.^5^ Furthermore, studies have shown that the serum levels of TNF-α are notably elevated in patients with SpA in comparison to individuals without the condition.^6^

In the past two decades, a new successful class of antirheumatic drugs has emerged: biologic disease-modifying antirheumatic drugs (bDMARDs), including tumour necrosis factor α inhibitors (TNFα-I). These drugs have helped to overcome the disappointment with the limited effectiveness of existing treatments for inflammatory rheumatic diseases. With the introduction of these innovative treatments, expectations have been raised, but not all issues have been fully resolved.

Despite the fact that the advent of biotherapies such as TNFα-I has favourably altered and revolutionised the treatment of patients with SpA which represents a therapeutic breakthrough, its maintenance is not always guaranteed because a significant proportion of SpA patients discontinued. The reasons why some patients continue with the same TNFα-I agent while others discontinue it remain unclear. Therefore, identifying the reasons and predictive factors for treatment discontinuation can assist in maintaining appropriate treatment regimens. Consequently, our study aims not only to analyse therapeutic maintenance but also to perceive the reasons and predictive factors of stopping TNFα-I as well.

## MATERIALS AND METHODS

### RBSMR Study

The registry of biotherapies of the Moroccan society of rheumatology (RBSMR) is a historical-prospective multicentre registry that includes multiple rheumatology departments from ten different university medical centres, encompassing university hospitals in Marrakech, Rabat, Agadir, Oujda, Casablanca, Fes, and military university hospitals in Agadir, Marrakech, Rabat, and Meknes. Patients recruited in the registry were over the age of 18 years, diagnosed with SpA and treated with biotherapy, either as an initiation or ongoing treatment, within Moroccan university medical centres. These patients provided written informed consent to participate in the registry. The inclusion period extended from June 2017 to January 2019, and the follow-up lasted for three years. A first database freeze occurred at the end of the inclusion period in January 2019. The primary objective of the registry was to evaluate the tolerability of biotherapy in rheumatology for patients with SpA. The second objective was to assess the effectiveness of biotherapies in rheumatology on patient’s quality of life as well as to monitor and evaluate patient management methods in real life.

### Study Aims

A national prospective multicentric cohort and analytical study was conducted using the baseline data of the RBSMR. In the present study, the following information was recorded from the patient’s registry:

Age, gender, ethnic origin.
Clinical, radiological, and biological characteristics of the population.
Comorbidities, extra-articular involvement, disease activity and therapies in this community.

Maintenance was defined as “the interval between the introduction and the suspension of the same TNFα-I”, and the reasons for discontinuation were analysed and divided into: undesirable effects, ineffectiveness and other reasons.

### Statistical Analysis

The statistical analysis for this study was performed using an SPSS (Statistical Package for the Social Sciences) software version 20. It proceeded through several steps to explore the therapeutic maintenance, the factors associated with it, comparing maintenance across different TNFα-I, and examining reasons and predictive factors for TNFα-I discontinuation.

In the descriptive analysis, quantitative variables were represented by means and standard deviations, while qualitative variables were expressed as percentages.

Subsequently, univariate analysis was employed to identify factors associated with therapeutic maintenance and TNFα-I discontinuation at each follow-up period. This analysis used the chi-square test for qualitative variables and student’s t-test for quantitative variables. Finally, multivariate analysis was conducted using binary logistic regression to simultaneously explore the effect of multiple variables on therapeutic maintenance and treatment discontinuation while controlling for the effects of other variables.

Parameters with a p-value ≤0.05 were considered statistically significant.

The treatment maintenance analysis was conducted using the Kaplan–Meier method.

## RESULTS

A total of 194 patients under biotherapy for SpA were included, of which 190 patients (97.9%) under TNFα-I. The follow-up period extended for three years. Their average age was 40.2 +/−13.6 years. The male gender predominated, with a male-to-female ratio of 1.71:1. On average, the duration of the disease was 11.7 +/−6.7 years, and the mean bath ankylosing spondylitis disease activity index (BASDAI) was 4.8. The involvement in our population was axial in 95.3%, peripheral in 67.7% (among them 56.4% on conventional disease-modifying antirheumatic drugs [cDMARDS]), and enthesitis in 58.3% of cases. 87.9% of patients had radiological sacroiliitis, 64.1% were on non-steroidal anti-inflammatory drugs (NSAIDs), and 56.4% on cDMARDS (**Table 1**).

**Table 1.** Characteristics of included patients (baseline data).

	No: 190
Age* (Year)	40.2+/−13.6
Axial involvement**	95.3%
Peripheral involvement** (Arthritis, dactylitis)	67.7%
Enthesitis**	58.3%
Anterior uveitis**	13.7%
Psoriasis**	6.7%
IBD**	10.9%
Radiographic sacroiliitis**	87.9%
Use of NSAIDs**	64.1%
cDMARDs**	56.4%

* Mean and standard deviation;

** Percentage.

IBD: inflammatory bowel disease; NSAIDs: nonsteroidal anti-inflammatory drugs; cDMARDs: conventional disease-modifying antirheumatic drugs.

**Figure 1.**
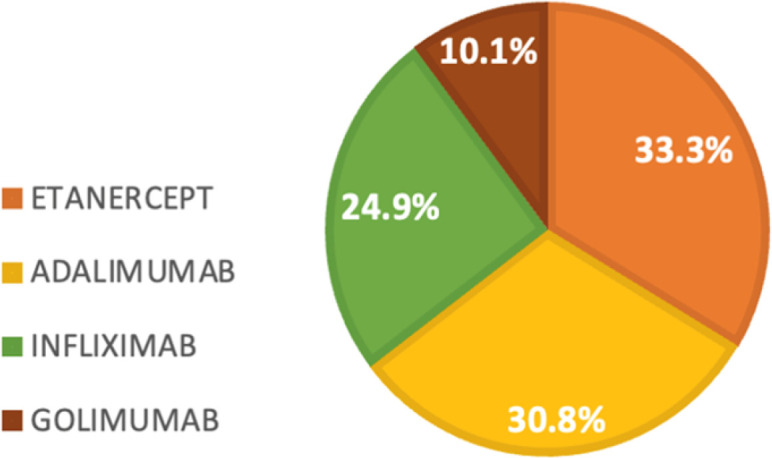
Distribution of patients based on the type of the TNFα-I utilised (%) at baseline.

**Figure 2.**
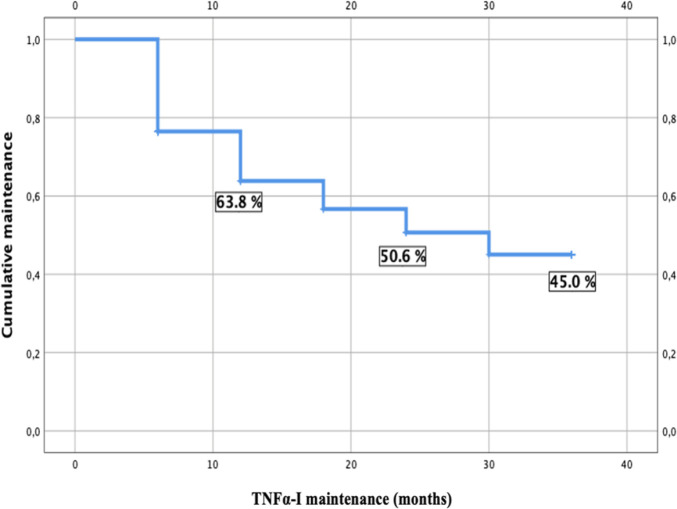
Kaplan-Meier curve of TNFα-I Global maintenance.

Four types of TNFα-I (bio-originators) were studied: etanercept 50mg/week subcutaneously, golimumab 50 mg/month subcutaneously, adalimumab 40mg/15 days subcutaneously and the infliximab 5mg/kg infused at weeks 0, 2, and 6, then every 8 weeks thereafter. Notably, none of the patients in our series were receiving certolizumab. The most widely used TNFα-I at the time of inclusion to the registry was etanercept which was used in approximately 33.3% of cases. This was followed by adalimumab, prescribed in 30.8% of cases, infliximab in 24.9% of cases, and golimumab in 10.1% of cases (**Figure 1**). The mean duration of maintenance for all TNFα-I combined was 23.5 months [(95% CI 21.6–25.4)]. The therapeutic maintenance decreased over time from 63.8% at 1 year to 45% at 3 years (**Figure 2**).

The maintenance of each TNFα-I was assessed over the whole study and it decreased over time. The mean duration of adalimumab maintenance was the highest (24.8 months), followed by etanercept (23.9 months), and lastly, with equal duration, infliximab, and golimumab (21.8 months) (**Table 2**).

**Table 2.** Therapeutic maintenance according to the type of TNF-α I utilised.

TNFα-I	Mean duration of therapeutic maintenance (months)	Confidence interval [IC] 95%		One year maintenance (%)	Two years maintenance (%)	Three years maintenance (%)
		Lower bound	Upper bound			
Etanercept	23.9	20.709	27.136	62.6	55.3	43.4
Adalimumab	24.8	21.309	28.303	67.3	53.5	51.1
Infliximab	21.8	18.089	25.614	60.4	40.8	37.7
Golimumab	21.8	15.636	28.057	63.2	45.9	45.9

**Figure 3.**
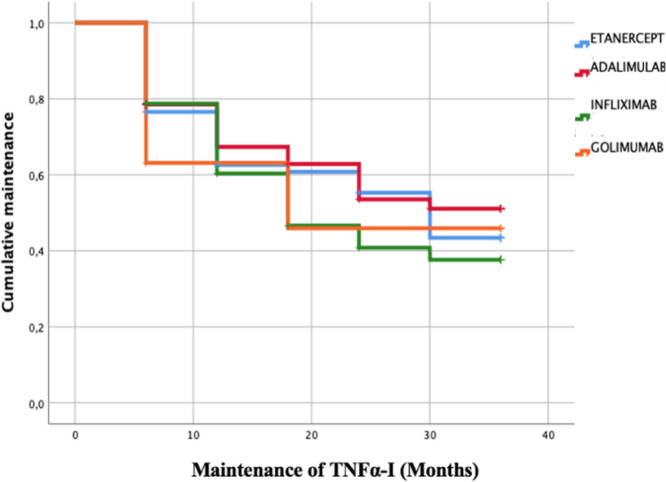
Kaplan-Meier curve of the maintenance therapeutic of each TNFα-I studied.

Furthermore, after 36 months of treatment, adalimumab exhibited the highest therapeutic maintenance (51.1%), followed by golimumab (45.9%), then etanercept (43.4%), and finally, infliximab (37.7%) (**Figure 3**).

**Figure 4.**
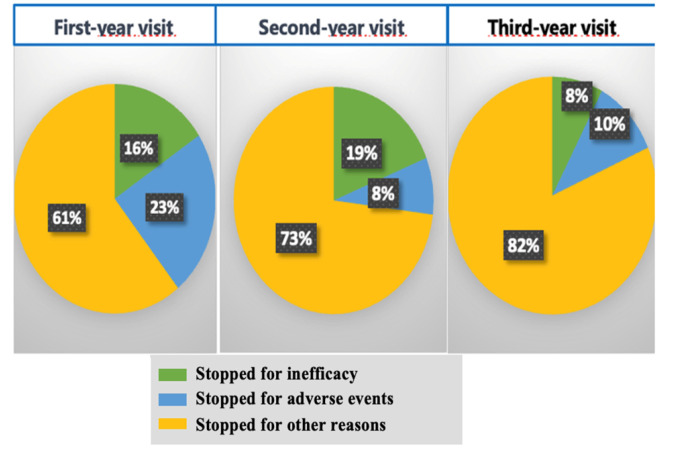
Causes of TNFα-I discontinuation at 1, 2, and 3 years of follow-up in %.

Both clinical efficacies and general safety profiles of bDMARDs more precisely the TNFα-I placed them at the top of rheumatologist’s preference; however, despite this tremendous progress made in the therapeutic management, some of them ended up stopping for several reasons (**Figure 4**), divided into adverse events, inefficacy and other causes (stockout of the drugs, COVID-19 lockdown, healthcare coverage, clinical-biological remission, the patient’s refusal to continue the treatment).

28.4% of our patients stopped their treatment at 1 year: 16% due to inefficacy, 23% for undesirable effects and 61% for various other reasons.

The discontinuation rate of treatment at 2 years was 22%: 19% for inefficacy, 8% for undesirable effects and 73% for other reasons.

After 3 years, 27.7% had stopped their treatment, with 8% citing ineffectiveness, 10% attributing it to undesirable effects, and 82% ceasing for various other reasons. Within this 82% who stopped for other reasons: 48.57% were due to the stockout of the utilized anti-TNF alpha, 14.28% were due to the COVID-19 lockdown, 17.14% halted due to clinical-biological remission, 5.71% resulted from the patient’s refusal to continue the treatment, and 2.9% were due to issues related to healthcare coverage. In the bivariate analysis, there was no association noticed between the type of the TNFα-I and therapeutic maintenance; whereas, the maintenance of treatment was significantly associated with many factors (**Table 3**).

**Table 3.** Results of bivariate analysis between the different types of TNFα-I and their therapeutic maintenance.

TNFα-I	Therapeutic maintenance at 1 year	Therapeutic maintenance at 2 years	Therapeutic maintenance at 3 years
Significant factors in bivariate analysis (P-value)	Satisfied PGA (p=0.012) Low BASMI (p=0.031) Average decrease in BASFI (p=0.011) VAS-pain = 0 (p<0.001) Absence of tuberculosis infection (p=0.049) BASDAI < 4 (p=0.006) CRP < 6mg/L (p=0.003)	Satisfied PGA (p=0.021) Low BASMI (p=0.013) Average decrease in BASFI (p=0.002) VAS-pain = 0 (p<0.001) Absence of plantar fasciitis (p=0.011) Absence of new comorbidities (p=0.05) BASDAI < 4 (p=0.01) Normal sedimentation rate (p=0.011) CRP < 6mg/L (p=0.001) NSAIDs not taken between and on the day of the visit (p=0.042), (p=0.015)	Satisfied PGA (p=0.021) Low BASMI (p=0.013) Average decrease in BASFI (p=0.002) VAS-pain = 0 (p<0.001) Absence of new comorbidities (p=0.05) Absence of infections (p=0.043) BASDAI < 4 (p=0.004) CRP < 6mg/L (p=0.022) NSAIDs not taken between visits (p=0.003)

PGA: patient global assessment; BASMI: bath ankylosing spondylitis metrology index; BASFI: bath ankylosing spondylitis functional index; BASDAI: bath ankylosing spondylitis disease activity index; CRP: C-reactive protein; VAS: visual analogue scale; NSAIDs: non-steroidal anti-inflammatory drugs.

- At 1 year with: Satisfied patient global assessment (PGA) (***p=0.012***), low bath ankylosing spondylitis metrology index (BASMI) (***p= 0.031***), average decrease in bath ankylosing spondylitis functional index (BASFI) (***p = 0.011***), visual analogue scale VAS-pain = 0 (***p=0.032***), the absence of tuberculosis disease (***p=0.049***), bath ankylosing spondylitis disease activity index (BASDAI) < 4 (***p=0.006***) and with a C-reactive protein (CRP) < 6mg/L (***p=0.003***).
- At 2 years with: Satisfied PGA (***p=0.021***), low BASMI (***p= 0.013***), and average decrease in BASFI (***p = 0.002***), VAS-pain = 0 (***p <0.001***), absence of plantar fasciitis. (***p=0.011***), absence of new comorbidities (***p=0.05***), BASDAI < 4 (***p=0.01***), normal sedimentation rate (***p=0.011***), CRP < 6mg/L (***p=0.001***) and with NSAIDs not taken between and on the day of the visit (***p=0.042***).
- At 3 years with: Satisfied PGA (***p<0.001***), BASDAI <4 (***p=0.004***), a CRP < 6mg/L (***p=0.022***), low BASMI (***p= 0.004***), average decrease in BASFI (***p=0.013***), VAS-pain = 0 (***p=0.017***), the absence of infections (***p =0.043***), Absence of new comorbidities (***p=0.02***) and NSAIDs not taken between visits (***p=0.003***).

The multivariate analysis of therapeutic maintenance did not reveal any correlation in the logistic regression.

As far as stopping the use of TNFα-I concerned, a multivariate analysis showed that the predictive factors for therapeutic discontinuation were associated with:

At 1 year with: the absence of analgesic use [Odds Ratio (OR) = 3.59; Confidence interval CI 95% (1.30–9.9)] (p-value:0,013) and CRP > 6 mg/L [OR = 2.74; CI 95% (1.17–6.41)](p-value:0,02).

At 2 years with: Taking NSAIDs between visits [***OR = 2.78; CI 95% (1.16–6.65***)], (p-value:0,021) and a BASDAI > 4 [***OR =1,47; CI 95% (1,14–1,90***)] (p-value:0,003).

At 3 years with: Taking NSAIDs between visits [***OR= 5.42; CI 95% (1.40–20.97)***] (p-value:0,014) and a high average ankylosing spondylitis disease activity score with C-reactive protein (ASDAS CRP) on the day of the visit [***OR= 2.84; CI 95% (1.43–5.63)***] (p-value:0,003).

## DISCUSSION

In our study, 190 patients with SpA under TNFα-I were followed for a mean of 36 months. The mean disease duration was 11.7 +/− 6.7 years. Patient characteristics were consistent with published data regarding SpA. Our most commonly used TNFα-I was etanercept (33.3% of cases), followed by adalimumab, infliximab and lastly golimumab. The ongoing therapeutic maintenance of all TNFα-I drugs in our study, when considered collectively, was relatively high but exhibited a decline over time, decreasing from 63.8% at the end of the first year to 45% after 3 years. It was not influenced by the specific type of TNFα-I, whether they were monoclonal antibodies or soluble TNFα receptors, which is in line with the results of nationwide registries in many other countries.^7^

However, it is noteworthy that within the 3-year follow-up period, therapeutic maintenance was significantly associated with several factors, including a Satisfied PGA, improved mobility and daily activities without discomfort, enhanced autonomy, the absence of pain and depression, the absence of new comorbidities, the absence of infections, BASDAI score <4, CRP levels below 6mg/L, and non-use of NSAIDs between visits. These results are close to those found in an Algerian study,^8^ where etanercept was the most prescribed treatment and demonstrated the best maintenance rates. The clinical response to treatment was sustained over 3 years and was associated with a high therapeutic maintenance rate of 72.5%. This implies a favourable trend in the efficacy and sustained effectiveness of etanercept in the treatment of SpA, consistent with broader clinical evidence. The predominant utilisation of etanercept in Maghrebian countries, where tuberculosis is endemic, may be elucidated by a reduced risk of tuberculosis reactivation observed in patients using etanercept as opposed to other TNFα-I.^9^

Nevertheless, the data in the literature are inconsistent on this point. As suggested by some published studies,^7,10^ where the adalimumab constituted the most commonly prescribed TNFα-I. Maureen Dubreuil et al. conducted a study involving 2437 patients with SpA, of whom 2322 (95.3%) received TNFα-I treatment and were monitored for up to 12 months. Adalimumab was the most frequently prescribed TNF α-I (55.8%) across all patients, followed by etanercept (22.7%). The rate of persistence after 12 months was estimated to be 56.4%.^10^

Faustine Krajewski et al. had carried out a study of 244 patients diagnosed with SpA followed for an average duration of 32 months from the initiation of their first TNF α-I treatment. The mean duration of the initial TNFα-I therapy was 21.7 months. Notably, the most commonly prescribed first TNFα-I, in decreasing order of frequency, was adalimumab, etanercept, golimumab, and infliximab. The prescribing pattern was influenced by the widespread preference for sub-cutaneous formulations as the primary option in France, mainly driven by economic considerations and established clinical practices.^7^ In the Czech National Registry ATTRA,^11^ a study by K. Pavelka et al. were conducted involving 310 patients diagnosed with SpA undergoing treatment with TNFα-I. The observed persistence rates for the initial TNFα-I were higher than our study and the literature, reaching 84% at the end of the first year, 76% after 2 years, and 72% following 3 years of treatment. Our study showed that in the context of treatment discontinuation at the 36-month follow-up, 27.7% had stopped their treatment: of which 8% for ineffectiveness, 10% undesirable effects, and 82% for other reasons. It was found that taking NSAIDs between visits and having a high average ASDAS CRP on the day of the visit were identified as predictive factors for therapeutic discontinuation in multivariate analysis. These factors played a role in influencing treatment discontinuation during the study.

In a French study,^7^ the foremost reason for discontinuation of the first TNFα-I was secondary lack of efficacy, accounting for 44% of cases, followed by primary lack of efficacy, which represented 38% of the reasons for treatment discontinuation. While within the ATTRA registry, significant risk factors for discontinuation of treatment were female gender (RR 2.22, p=0.001) and having elevated C-reactive protein (CRP) levels (RR 1.33, p=0.025). The proportion of patients with BASDAI <4 during the treatment period was higher in the etanercept group than in the infliximab group (p<0.001).^11^ In the study conducted by Gulyas et al. which involved 175 patients with SpA under treatment with TNFα-I. It was observed that older age at the initiation of the first TNFα-I [mean age 42.5 (SD 12.6) versus 38.8 (SD 11.2) years] was identified as a risk factor for discontinuation.^12^

One notable limitation of our study is the potential lack of generalisability of the results. A significant proportion of patients discontinued TNFα-I for reasons unrelated to their medical condition. Despite efforts to account for various confounding factors, the influence of non-medical reasons for discontinuation on the observed outcomes cannot be overlooked. Furthermore, efforts to enhance patient education and support programs may play a crucial role in promoting treatment adherence and mitigating non-medical reasons for discontinuation. By empowering patients with comprehensive information about their condition, treatment options, and potential benefits of therapy, healthcare providers can foster a collaborative approach to decision-making and facilitate shared treatment goals. Additionally, initiatives aimed at addressing socioeconomic disparities and improving access to healthcare services may help alleviate barriers to treatment adherence and reduce the likelihood of non-medical discontinuation.

## CONCLUSION

The therapeutic maintenance level of TNFα-I in our study was satisfactory and comparable to other studies. Our prospective multicentric cohort and analytical study provides a more comprehensive understanding of the factors that contribute to the improved maintenance of treatment with TNF-alpha inhibitors. It delves into the reasons influencing treatment continuity and identifies predictive factors associated with discontinuation, which ultimately lead to the cessation of therapy.

## Data Availability

The datasets are available from the RBSMR registry of the Moroccan Society of Rheumatology.
